# A Synchronous Gastric Intramural Metastasis from Multiple Gastric Cancers

**DOI:** 10.70352/scrj.cr.25-0071

**Published:** 2025-06-11

**Authors:** Daisuke Horikawa, Hiroki Takahata, Yasuhiro Fujiwara

**Affiliations:** Department of Surgery, Furano Kyokai Hospital, Furano, Hokkaido, Japan

**Keywords:** intramural metastasis, gastric intramural metastasis, gastric cancer, multiple gastric cancer

## Abstract

**INTRODUCTION:**

Although intramural metastasis has been reported in esophageal cancer, it is extremely rare in gastric cancer. In this report, we describe a case of gastric intramural metastasis in a patient with multiple gastric cancers.

**CASE PRESENTATION:**

A 49-year-old woman was referred to our hospital after abnormalities were detected during barium screening. Computed tomography revealed gastric wall thickening with some areas of calcification and enlargement of the lesser curvature of the lymph nodes. Upper gastrointestinal endoscopy identified the following: (1) a type 2 tumor (sig-muc-tub1) on the posterior wall of the gastric angle, (2) a type 2 tumor (muc) on the anterior wall of the lower gastric body, and (3) a 0-IIc lesion (tub1) on the posterior wall of the middle gastric body. Additionally, (4) a submucosal tumor-like elevation with erosion was noted on the greater curvature of the gastric angle; however, no cancer cells were detected in this lesion. Given the patient's young age and the presence of multiple lesions, laparoscopic total gastrectomy with D2 lymphadenectomy was performed. The postoperative course was uneventful, and the patient was discharged on postoperative day 10. Postoperative pathological examination confirmed that lesions (1)–(3) were gastric cancers, staged as pT2N1M0, Stage IIA. However, lesion (4) showed no cancer cells in the mucosal layer but demonstrated mucinous carcinoma in the submucosal layer, which was diagnosed as gastric intramural metastasis. Based on the Japanese gastric cancer guidelines, postoperative adjuvant chemotherapy with S-1 was planned. At 4 months postoperatively, the patient is undergoing adjuvant chemotherapy and remains recurrence-free.

**CONCLUSIONS:**

We encountered a case of gastric intramural metastasis in a patient with multiple gastric cancers. Intramural metastasis in gastric cancer is extremely rare, and further accumulation of cases is essential to elucidate its clinicopathological features. We hope that this case report will contribute to the accumulation of cases and enhance our understanding of the characteristics of intramural metastasis in gastric cancer.

## Abbreviations

IM
: intramural metastasis
SMT
: submucosal tumor

## INTRODUCTION

IM in esophageal cancer is occasionally observed; however, IM in gastric cancer is extremely rare. While IM is recognized as a poor prognostic factor in esophageal cancer, it is also considered an indicator of poor prognosis in gastric cancer. However, studies on IM in gastric cancer remains limited. To our knowledge, only one study has examined IM in gastric cancer, which includes a substantial number of cases. However, in that study, only 29 cases of IM were documented, indicating the scarcity of such cases. Here, we present a case of synchronous solitary IM in a patient with multiple gastric cancers.

## CASE PRESENTATION

**Fig. 1:**
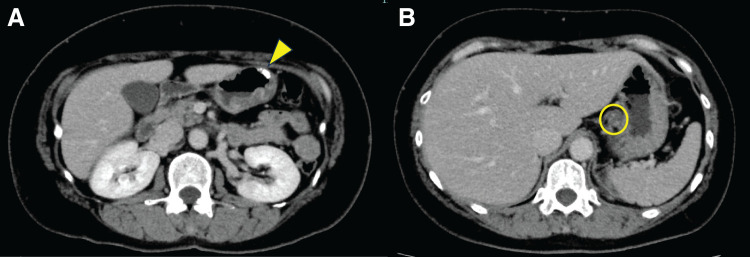
Preoperative CT imaging. (**A**) Wall thickening and partial calcification (arrowhead) are observed in the gastric body. (**B**) Enlargement of the lesser curvature lymph node suggesting lymph node metastasis (circle).

**Fig. 2:**
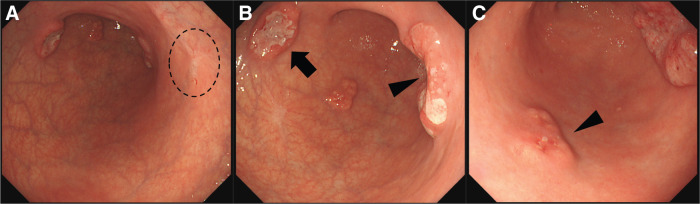
Preoperative upper gastrointestinal endoscopy. (**A**) The most proximal lesion (dotted circle, corresponding to lesion 3 in the text) is observed as a 0-IIc lesion on the posterior wall of the middle gastric body. (**B**) Type 2 tumors are observed on the posterior wall of the angular region (arrowhead, corresponding to lesion 1 in the text) and the anterior wall of the lower gastric body (arrow, corresponding to lesion 2 in the text). (**C**) A submucosal tumor-like elevation with surface erosion (arrowhead, corresponding to lesion 4 in the text) is observed in the greater curvature of the angular region.

A 49-year-old woman was referred to our hospital after the detection of abnormalities during barium screening. CT revealed wall thickening in the gastric body with partial calcification. Additionally, enlargement of the lesser curvature lymph nodes (No. 3) was observed, raising suspicion of metastasis (**Fig. 1**). No distant metastases were detected in the liver, lungs, or any other organs. Upper gastrointestinal endoscopy revealed 4 distinct gastric lesions. The biopsy findings were as follows: (1) a Type 2 tumor on the posterior wall of the gastric angle, diagnosed as a sig-muc-tub1; (2) a Type 2 tumor on the anterior wall of the lower gastric body, diagnosed as muc; (3) Type 0-IIc lesion on the posterior wall of the middle gastric body, diagnosed as tub1; and (4) a 15 mm SMT like elevated lesion with erosion on the greater curvature of the gastric angle in which no malignant cells were detected (**Fig. 2**).

Given the patient's relatively young age and the presence of multiple lesions, we decided, in consultation with the patient, to perform laparoscopic total gastrectomy with D2 lymph node dissection, Roux-en-Y reconstruction, and prophylactic cholecystectomy. The operation lasted for 260 minutes, with an estimated blood loss of 22 mL. The postoperative course was uneventful, and the patient was discharged on the 10th postoperative day.

**Fig. 3:**
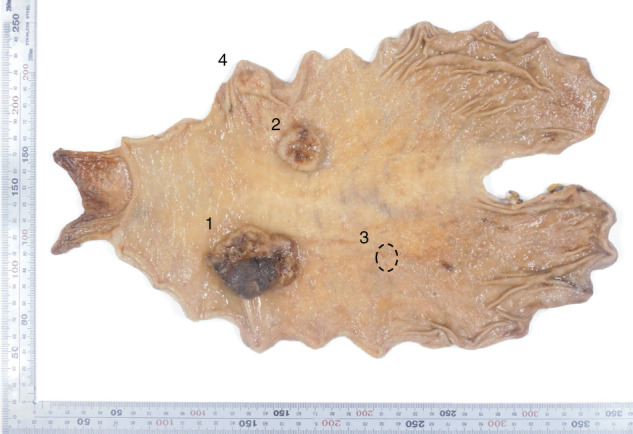
Postoperative histopathological findings—gross specimen. (1) L, Post, Type2, 55 × 40 mm, muc >tub2 >tub1, pT2, INFa, Ly1a, V0, pPM0 (120 mm), pDM0 (85 mm), pN1 (1/61), cM0, CY0, pStageIIA; (2) M, Ant, Type2, 25 × 25 mm, muc, pT2 (MP), INFa, Ly1a, V0, pPM0 (95 mm), pDM0 (120 mm); (3) (dotted circle) M, Post, Type 0-IIc, 10 × 9 mm, tub1, pT1a (M), Ly0, V0, pPM0 (65 mm), pDM0 (180 mm); (4) L, Gre, 20 × 13 mm, mucinous adenocarcinoma in the submucosa without cancer cells in the mucosa, diagnosed as intramural metastasis.

**Fig. 4:**
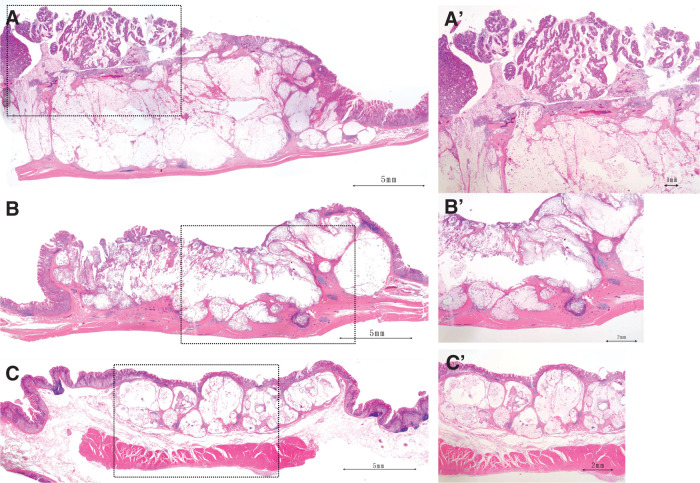
Postoperative histopathological findings—microscopic examination. (**A**) (lesion 1): The histological type is muc >tub2 >tub1, and the tumor has invaded the muscularis propria. **A’** (dotted area of **A**): Tub2 > tub1-type adenocarcinoma is observed in the mucosal layer. (**B**) (lesion 2): The histological type is mucinous adenocarcinoma (muc), and the tumor has invaded the muscularis propria. **B’** (dotted area of **B**): Normal mucosa is partially preserved, and no apparent tubular adenocarcinoma is observed in the mucosal layer. While both synchronous multiple gastric cancers and intramural metastasis are possible, a definitive diagnosis is difficult to establish. (**C**) (lesion 4): The histological type is mucinous adenocarcinoma (muc), and the tumor is confined to the submucosal layer. **C’** (dotted area of **C**): The mucosa is histologically normal, and mucinous adenocarcinoma is confined to the submucosa.

Postoperative histopathological examination revealed the following findings: (1) L, Post, Type 2, 55 × 40 mm, muc >tub2 >tub1, pT2 (MP), INFa, Ly1a, V0, pPM0 (120 mm), pDM0 (85 mm); (2) M, Ant, Type 2, 25 × 25 mm, muc, pT2 (MP), INFa, Ly1a, V0, pPM0 (95 mm), pDM0 (120 mm); (3) M, Post, Type 0-IIc, 10 × 9 mm, tub1, pT1a (M), Ly0, V0, pPM0 (65 mm), pDM0 (180 mm); and (4) 20 × 13 mm, mucinous adenocarcinoma in the submucosa without cancer cells in the mucosa, diagnosed as intramural metastasis (**Figs. 3** and **4**). Lesion numbers correspond to those described previously. Regarding capillary invasion in intramural metastatic lesions, only lymphatic invasion (Ly1a) was observed, with no evidence of venous invasion (V0). Additional findings included pN1 (1/61), cM0, R0, CY0, and pStage IIA.

Based on the Japanese Gastric Cancer Treatment Guidelines, the patient was scheduled to undergo adjuvant chemotherapy with S-1. At 4 months postoperatively, the patient is undergoing adjuvant chemotherapy and remains recurrence-free.

## DISCUSSION

Reports of IM in gastric cancer are extremely limited. Hashimoto et al.^1)^ analyzed 4,714 cases of gastric cancer and identified only 29 cases (0.6%) of IM, highlighting the rarity of this condition. In contrast, IM in esophageal cancer is reported to occur with a higher frequency, ranging from 10.8% to 15.2%.^2–4)^

The diagnostic criteria for IM in gastric cancer include (1) clear separation from the primary tumor; (2) location in the wall of the esophagus, stomach, or duodenum; (3) gross appearance of a submucosal tumor without intraepithelial cancer extension; (4) the same histological type as the primary tumor; and (5) lack of evidence of intravascular growth.^1)^ All of these criteria were met in lesion 4 of our case. On the other hand, lesion 2 consisted solely of mucinous adenocarcinoma and lacked any adenocarcinoma component in the mucosal layer. Therefore, the possibility that this lesion also represents intramural metastasis cannot be entirely ruled out. However, because it shares a common histological type with lesion 1, the possibility of synchronous multiple gastric cancers must also be considered. Consequently, it remains difficult to definitively distinguish whether these lesions represent intramural metastasis or multiple primary tumors.

In all reported cases of IM in gastric cancer, the primary tumor was classified as T2 or deeper, with capillary invasion (lymphatic or vessel) and lymph node metastasis. The mean size of the IM is approximately 2 cm, and it is found at various levels from the submucosa to the serosa^1)^. The preoperative diagnostic rate of IM has been reported to be 17.2%,^1)^ underscoring its diagnostic challenge. In contrast, the preoperative diagnostic rate of IM in esophageal cancer is relatively high (54.2%), with lesions often presenting as dome-shaped SMT covered by normal epithelium and frequently accompanied by erosion or ulceration.^2)^ The distance between the primary lesion and the site of the IM is typically reported to be within 2 cm. However, IM have been observed as far as 5.5 cm away, with some cases involving the duodenum or esophagus.^1,5)^ IM of gastric and esophageal cancer is thought to occur via lymphatic invasion.^1,6)^ In our case, the macroscopic appearance of the IM was a 20 × 13 mm SMT-like elevated lesion with erosion. Histopathological findings showed T2, lymphatic invasion (Ly1a), and lymph node metastasis (N1) with distances of 15 mm and 30 mm from the suspected primary lesions (2) and (3), respectively, which were consistent with previous reports. Additionally, the presence of lymphatic invasion and positive lymph node metastasis suggested that intramural metastasis developed through the lymphatic flow.

Patients with esophageal cancer and IM have an extremely poor prognosis, with a 5-year survival rate of 9% and a median survival time of 7 months.^3)^ In gastric cancer, IM is similarly associated with poor outcomes, with a reported 3-year survival rate of 13.9% and a median survival time of 11 months. In comparison, patients with advanced gastric cancer without IM have a median survival time of 39.4 months and a 3-year survival rate of 51.1%,^1)^ confirming that IM is a significantly poor prognostic factor in gastric cancer. However, in cases of gastric cancer with IM, where the distance from the primary lesion is less than 1 cm and R0 resection is achieved, the prognosis is notably better, with a reported 3-year survival rate of 41.7% and a median survival time of 29 months.^1)^ In our case, considering the potential for a poor prognosis, careful postoperative follow-up is necessary. As the distance between the primary lesion and IM is approximately 2 cm in most cases, the importance of securing adequate resection margins, as recommended in the Japanese gastric cancer treatment guidelines, is emphasized. Furthermore, when SMT-like features are observed, the possibility of IM should be considered. This highlights the importance of selecting the appropriate surgical techniques to ensure the inclusion of such lesions in the resection field.

## CONCLUSIONS

We report a case of IM in a patient with multiple gastric cancers. IM in gastric cancer is extremely rare, and further accumulation of cases is essential to elucidate its clinicopathological features. We hope that this case report will contribute to the accumulation of cases and enhance our understanding of the characteristics of IM in gastric cancer.

## DECLARATIONS

### Funding

Not applicable.

### Authors’ contributions

DH determined the treatments.

DH, HT, and YF performed the surgery and participated in the patient treatment.

All the authors have read and approved the final version of the manuscript.

### Availability of data and materials

Not applicable.

### Ethics approval and consent to participate

Not applicable.

### Consent for publication

Informed consent for publication was obtained from the patient described in this case report, and the identity was protected.

### Competing interests

The authors declare that there are no conflicts of interest.
